# Association Between Tooth Eruption and Parent‐Reported Awake Bruxism in Early Childhood

**DOI:** 10.1111/joor.14048

**Published:** 2025-06-04

**Authors:** Letícia Fernanda Moreira‐Santos, Caio Rafael Schavarski, Cássia Cilene Dezan‐Garbelini, Ivana Meyer Prado, Isabela Almeida Pordeus, Saul Martins Paiva, Júnia Maria Serra‐Negra

**Affiliations:** ^1^ Departament of Pediatric Dentistry, School of Dentistry Universidade Federal Minas Gerais Belo Horizonte Minas Gerais Brazil; ^2^ Department of Oral Medicine and Paediatric Dentistry Universidade Estadual de Londrina Londrina Paraná Brazil

**Keywords:** behaviour, bruxism, child, child development, primary tooth, tooth eruption

## Abstract

**Background:**

Mandibular instability during primary tooth eruption has been suggested as a factor associated with parent‐reported awake bruxism (PRAB) in early childhood, but this relationship remains unclear.

**Objective:**

To evaluate the association between PRAB frequency and the number of erupted primary teeth by dental groups, as well as signs and symptoms of tooth eruption, oral habits, and sociodemographic characteristics.

**Methods:**

This cross‐sectional study included 144 Brazilian children aged 4–36 months. Parents/caregivers completed an interview‐based questionnaire covering sociodemographic characteristics, oral habits, signs and symptoms of tooth eruption, and PRAB frequency. A single‐trained dentist performed oral examinations to record erupted primary teeth. Descriptive analysis, chi‐square, and Kruskal‐Wallis tests were conducted (*p* < 0.05).

**Results:**

The prevalence of PRAB was 16.7%. No significant association was found between PRAB frequency and sex, age, oral habits, or signs and symptoms of tooth eruption. However, more erupted primary molars (*p* = 0.038) and canines (*p* = 0.022) were associated with a lower PRAB frequency.

**Conclusion:**

The number of erupted primary molars and canines was associated with the frequency of PRAB in early childhood, supporting the hypothesis that AB during this stage may represent an adaptive behaviour.

## Graphical abstract

Graphical abstract illustrating the relationship between tooth eruption and parent‐reported awake bruxism in early childhood.

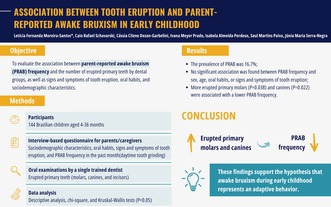

## 1 Background

Bruxism is a behaviour characterised by masticatory muscle activity with two distinct circadian manifestations: sleep bruxism (SB), which occurs during sleep, and awake bruxism (AB), which occurs during wakefulness. In addition to tooth contact, bruxism may also involve mandiular bracing and/or thrusting [1]. A comprehensive assessment of bruxism may require integrating subject‐reported, clinical, and instrumental data to enhance diagnostic accuracy and management [2].

Bruxism has been observed as early as a median age of 10.5 months [3]. A recent systematic review indicated that the prevalence of AB is approximately 18% among children up to 10 years old and 19% among adolescents aged 10–19 years [4]. Among schoolchildren, AB has been associated with electronic device use and experiences of bullying [5, 6]. The literature also highlights a link between AB and emotional symptoms, such as stress and anxiety [7].

Traditionally, bruxism has been linked to adverse outcomes, including headaches, temporomandibular joint disorders, tooth wear, and fractures [1, 8, 9]. However, recent perspectives suggest that bruxism may also serve physiological functions [10]. The concept of bruxism as a “harmless behaviour” proposes potential benefits, such as maintaining airway patency [10].

In early childhood, the absence of a fully developed occlusion may lead to mandibular instability, resulting in involuntary jaw movements and tooth grinding, which parents/caregivers may interpret as bruxism [11]. Additionally, tooth eruption‐related tissue changes, such as inflammation and swelling, may contribute to discomfort [12]. Infants might grind their teeth reflexively in response to these symptoms, potentially alleviating irritability and itchy gums [13, 14, 15]. Consequently, bruxism during this developmental phase may represent an adaptive behaviour rather than a pathological condition.

These insights highlight the need for a nuanced approach to bruxism assessment across different age groups [11, 16]. Despite growing interest in this topic, research on bruxism in early childhood—a period of significant growth and neuromuscular development— [17] remains limited. Clarifying whether bruxism in young children is an adaptive response or an indicator for clinical intervention is essential [10]. A deeper understanding of its relationship with tooth eruption could provide valuable guidance for oral health professionals, aiding in the development of appropriate monitoring and management strategies tailored to young children.

Therefore, this study aimed to evaluate the association between the number of erupted primary teeth and the frequency of parent‐reported awake bruxism (PRAB) in early childhood, focusing on daytime tooth grinding. It also explored associations with sociodemographic characteristics, oral habits, signs, and symptoms of tooth eruption.

## 2 Methods

The study was conducted following the ethics principles outlined in the Declaration of Helsinki and received approval from the Research Ethics Committee of *Universidade Estadual de Londrina*, Brazil (protocol #4.479.888).

### 2.1 Study Design, Setting, and Sample

This cross‐sectional study was conducted in Londrina, southern Brazil, from September 2021 to April 2022. The study adhered to the Strengthening the Reporting of Observational Studies in Epidemiology (STROBE) guidelines [18]. Participants were children regularly attending the Child Oral Health Program at a dental clinic affiliated with *Universidade Estadual de Londrina*.

The program provides continuous monitoring for children from 3 months to 6 years old, with appointment frequency based on caries risk assessment. Eligible participants included children aged four to 36 months, of both sexes, with or without complete primary dentition. Exclusion criteria comprised children using anticonvulsant medications or those diagnosed with syndromes, developmental disorders, or cognitive impairments [5, 6]. Health and medication information was obtained from reports provided by parents/caregivers.

### 2.2 Data Collection

Data collection occurred during routine appointments at the children's dental clinic. A single‐trained dentist conducted interviews with parents/caregivers and performed oral examinations on the children before their scheduled dental visits. Parents/caregivers were informed about the study's objectives and risks in the waiting room, and those who agreed to participate provided written informed consent.

A questionnaire with 14 questions was administered through an interview lasting approximately 10 min. Four questions addressed sociodemographic characteristics, including the child's sex, age, and health status. Nine questions evaluated the presence of oral habits (e.g., pacifier use, digit sucking, nail‐biting, and object biting), as well as signs and symptoms of tooth eruption (e.g., irritability, itchy gums, increased salivation, and loss of appetite) [14]. Responses were recorded as either “no” or “yes”.

Parents/caregivers also reported PRAB frequency using an internationally accepted diagnostic approach [1, 19]. The key question was formulated as follows:

1. In the past month, how often have you noticed your child grinding their teeth while awake?

Response options included: “Not once”, “At least once a week”, “Twice a week”, and “Three times or more times a week”. PRAB frequency was categorised as follows: “absent”, “once or twice per week”, and “three or more times per week” [1, 19].

Oral examinations were performed under artificial light using a mouth mirror, with the child positioned on a paediatric examination stretcher. All biosafety protocols were followed. The presence or absence of each tooth was recorded in an odontogram. A tooth was considered erupted when an incisal edge or a molar cusp was visible or palpable through the gum [13].

### 2.3 Pilot Study

A pilot study was conducted with 23 children aged four to 36 months, all enrolled in the Child Oral Health Program. The objective was to assess the feasibility of the methodology and establish parameters for sample size calculation. Since no modifications to the method were deemed necessary, the pilot study participants were included in the main study.

### 2.4 Sample Size

Sample size calculation was based on the mean number of erupted teeth in children whose parents/caregivers reported no PRAB episodes (17.0; ±3.1) compared to those whose parents/caregivers reported PRAB frequency one to two times per week (13.0; ±8,0) in the pilot study. Considering an 80% statistical power and a 95% confidence level, the minimum sample size was calculated to be 44 children.

### 2.5 Data Analysis

Statistical analyses were performed using SPSS software (version 21.0, IBM, Armonk, NY, USA). Descriptive statistics were used to assess variable distributions and characterise the sample. Signs and symptoms of tooth eruption were consolidated into a single variable and classified as “present” or “absent.” This classification was based on whether parents/caregivers reported at least one of the following: irritability, itchy gums, increased salivation, or loss of appetite.

Spearman's correlation test assessed the relationship between the child's age and the number of erupted primary molars and canines. Associations between PRAB frequency and categorical variables (child's sex, oral habits, and signs and symptoms of tooth eruption) were assessed using the chi‐square test (*p* < 0.05). Data normality was verified using the Shapiro–Wilk test. Non‐parametric analyses, including the Kruskal–Wallis test with pair‐wise comparisons (post hoc) with the Mann–Whitney test, were performed to examine relationships between quantitative independent variables and PRAB frequency (*p* < 0.05). For the post hoc analysis, three pair‐wise comparisons were carried out: (1) comparison between children without PRAB (absent), (2) between children with PRAB activities once or twice per week, and (3) between children with PRAB three or more times per week. Bonferroni's correction was applied to minimise a type I error of incorrectly rejecting the null hypothesis due to three pair‐wise comparisons. The significance level (*α*) of 0.05 was divided by three, and the statistical significance for each pair‐wise comparison using the Mann–Whitney test was set at *p* < 0.017.

## 3 Results

All invited parents/caregivers agreed to participate in the study and completed the questionnaire in full. The final sample included 144 children with a mean age of 23.3 months (±9.9). The mean number of erupted primary molars, canines, and incisors was 4.0 (±3.0), 2.4 (±1.9), and 6.3 (±3.0), respectively. The prevalence of PRAB was 16.7% (Table 1).

**TABLE 1:** Sample characteristics (*n* = 144).

Quantitative variables	Mean (SD)
Child's age (months)	23.3 (±9.9)
Number of erupted primary molars	4.0 (±3.0)
Number of erupted primary canines	2.4 (±1.9)
Number of erupted primary incisors	6.3 (±3.0)

Categorical variables	*n* (%)
Child's sex	
Male	63 (43.8)
Female	81 (56.3)
Pacifier use	
No	109 (75.7)
Yes	35 (24.3)
Digit sucking	
No	123 (85.4)
Yes	21 (14.6)
Objects biting	
No	106 (73.6)
Yes	38 (26.4)
Nal‐biting	
No	131 (91.0)
Yes	13 (9.0)
Signs and symptoms of tooth eruption	
No	12 (8.3)
Yes	132 (91.7)
Parent‐reported awake bruxism	
Absent	120 (83.3)
Once or twice per week	9 (6.3)
Three or more times per week	15 (10.4)

Abbreviation: SD, standard deviation.

Spearman's correlation analysis revealed a significant positive correlation between age and the number of erupted primary canines (*r* = 0.768, *p* < 0.001) and primary molars (*r* = 0.757, *p* < 0.001).

Table 2 indicates no significant association between PRAB frequency and the child's sex, oral habits, signs, and symptoms of tooth eruption. Table 3 reveals that the number of erupted primary molars was significantly higher among children with parent‐reported awake bruxism once or twice per week (median = 8.0; min‐max = 4–8) when compared to children with awake bruxism activity three or more times per week (median = 2.7; min‐max = 2–7) (*p* = 0.004). Also, the number of erupted primary canines was significantly higher among children with awake bruxism frequency once or twice per week (median = 4.0; min‐max = 4–4) when compared to children with awake bruxism activity three or more times per week (median = 2.0; min‐max = 0–4) (*p* = 0.007).

**TABLE 2:** Distribution of the child's sex, oral habits, and signs and symptoms of tooth eruption by frequency of parent‐reported awake bruxism (*n* = 144).

	Parent‐reported awake bruxism *n* (%)			*p* ^a^
	Absent	Once or twice per week	Three or more times per week	
Child's sex				
Male	51 (81.0)	5 (7.9)	7 (11.1)	
Female	69 (85.2)	4 (4.9)	8 (9.9)	0.750
Pacifier use				
No	89 (81.7)	9 (8.3)	11 (10.1)	
Yes	31 (88.6)	0 (0)	4 (11.4)	0.238
Digit sucking				
No	103 (83.7)	7 (5.7)	13 (10.6)	
Yes	17 (81.0)	2 (9.5)	2 (9.5)	0.710
Object biting				
No	89 (84.4)	8 (7.5)	9 (8.5)	
Yes	31 (81.6)	1 (2.6)	6 (15.8)	0.318
Nail‐biting				
No	109 (83.2)	9 (6.9)	13 (9.9)	
Yes	11 (84.6)	0 (0)	2 (15.4)	0.701
Signs and symptoms of tooth eruption				
No	11 (91.7)	0 (0)	1 (8.3)	
Yes	109 (82.6)	9 (6.8)	14 (10.6)	1.000

^a^ Fisher test.

**TABLE 3:** Child's age and number of erupted primary teeth according to frequency of parent‐reported awake bruxism (*n* = 144).

	Parent‐reported awake bruxism				Pairwise comparisons		
	Absent	Once or twice per week	Three or more times per week	Kruskal–Wallis *p* ^a^	Absent versus once or twice per week *p* ^b^	Absent versus three or more times per week *p* ^b^	Once or twice per week versus three or more times per week *p* ^b^
Child's age (months)							
Mean (SD)	23.3 (±10.1)	28.1 (±5.2)	20.2 (±9.8)				
Median [Min − Max]	25.5 [4–36]	30.0 [18–35]	18.0 [6–35]	0.260			
Number of erupted primary molars							
Mean (SD)	4.0 (±3.0)	6.2 (±2.1)	2.7 (±2.6)				
Median [Min − Max]	4.0 [0–8]	8.0 [4–8]	2.7 [0–7]	0.025	0.039	0.122	**0.004**
Number of erupted primary canines							
Mean (SD)	2.4 (±1.9)	4.0 (±0)	1.9 (±1.9)				
Median [Min − Max]	4.0 [0–4]	4.0 [4–4]	2.0 [0–4]	0.022	0.024	0.272	**0.007**
Number of erupted primary incisors							
Mean (SD)	6.3 (±3.0)	8.0 (±0)	5.2 (±3.6)				
Median [Min − Max]	8.0 [0–8]	8.0 [8–8]	8.0 [0–8]	0.093			

*Note:* Bold values indicate statistically significant differences.

Abbreviations: Max, maximum; Min, minimum; SD, standard deviation.

^a^ Kruskal–Wallis test at *p* > 0.050.

^b^ Mann–Whitney test with Bonferroni correction with significance level set at *p* < 0.017.

## 4 Discussion

This study examined the association between PRAB frequency and the number of erupted primary teeth, along with signs and symptoms of tooth eruption, oral habits, and sociodemographic factors. Our findings indicate that PRAB frequency decreases as the number of erupted primary molars and canines increases, suggesting that jaw stabilisation during tooth eruption may reduce AB episodes [17]. This supports the hypothesis that AB in infants may serve an adaptive function rather than purely be detrimental.

Bruxism is centrally regulated, with studies demonstrating its dissociation from peripheral factors such as dental malocclusion [20, 21]. However, in infants, both the central nervous system and the stomatognathic system are still undergoing maturation [17]. The central incisors typically erupt between 8 and 12 months, lateral incisors between 9 and 18 months, first molars between 13 and 19 months, canines between 16 and 22 months, and second molars between 23 and 33 months [17]. Given this developmental process, tooth eruption influences jaw stabilisation and occlusion in young children [17].

Teething discomfort, often due to inflamed and swollen gums, may lead infants to grind their teeth reflexively [12]. This inflammatory response also stimulates salivation and has been associated with appetite loss, likely due to increased cytokine levels in the gingival fluid [12]. Interestingly, no significant association was found between these symptoms and PRAB frequency, possibly because parents actively manage teething discomfort. Studies suggest that caregivers frequently intervene to relieve teething distress [15], which may, in turn, affect PRAB reporting. Future research should explore whether symptom management influences AB episodes.

The PRAB prevalence in this study was consistent with a recent systematic review [4], but lower than the 56% prevalence reported in an earlier cross‐sectional study [3]. These discrepancies may stem from differences in diagnostic methods, distinctions between circadian manifestations of bruxism, and variations in study populations, including geographic and age‐related differences [5, 6, 16, 22, 23].

No significant associations were found between PRAB frequency and the child's sex or age, consistent with previous studies that have reported mixed findings on these variables [6, 23, 24]. Similarly, oral habits, such as pacifier use, digital sucking, nail‐biting, and object biting, were not linked to PRAB frequency. Although these behaviours may occur throughout the day, either consciously or unconsciously, they tend to influence mandibular activity primarily during sleep (i.e., sleep bruxism), which was not the main focus of this study. The lack of association between age and PRAB may reflect individual variability in tooth eruption timing, as well as the influence of behavioural and psychosocial factors beyond chronological age. Future studies on PRAB in early childhood should consider not only the number of erupted teeth but also incorporate assessments of behavioural and psychosocial dimensions. Longitudinal designs, in particular, may offer valuable insights into how these factors interact and evolve.

This study has limitations. PRAB frequency was based on parental reports of daytime tooth grinding‐an observable and well‐documented behaviour suitable for caregiver assessment [16]. However, mandibular bracing and thrusting in infants remain challenging to assess due to the difficulties of early observation. Future research should explore age‐appropriate methods to better understand AB in infancy. Although parental reports may introduce bias [6, 14], evaluating PRAB frequency rather than its mere presence may improve diagnostic accuracy [1, 24]. Notably, maternal–infant bonding, particularly in children under three, may mitigate reporting biases, as caregivers closely monitor their child's development [25].

Despite these limitations, this study provides a foundation for further research on AB in early childhood and offers valuable insights into its physiological role during stomatognathic system development. Our findings underscore the importance of considering age‐related factors when investigating AB in young children [11, 16]. Clinicians should monitor primary dentition development closely and recognise AB as a potentially adaptive behaviour. While often transient at this stage, it may serve a functional purpose but warrants attention if persistent. Given its multifactorial nature, a multidisciplinary approach is crucial, particularly when bruxism continues into later developmental stages [5, 6, 16, 22, 23].

## 5 Conclusions

The number of erupted primary molars and canines was associated with the frequency of PRAB in early childhood, supporting the hypothesis that AB during this stage may represent an adaptive behaviour.

## Author Contributions

Conceptualization, C.R.S., C.C.D.‐G., J.M.S.‐N., and I.A.P.; Data collection, C.R.S.; formal analysis, L.F.M.‐S., I.M.P and C.R.S.; writing and original draft preparation, L.F.M.‐S., I.M.P and C.R.S.; supervision, C.C.D.‐G., J.M.S.‐N., and I.A.P.; review and editing, L.F.M.‐S., C.R.S., C.C.D.‐G., I.M.P., J.M.S.‐N., and I.A.P.

## Ethics Statement

The study was conducted following the Declaration of Helsinki and approved by the Research Ethics Committee of *Universidade Estadual de Londrina*, Brazil (protocol #4.479.888).

## Consent

All parents/caregivers agreed to participate in the study and authorised their children's participation by signing a consent form.

## Conflicts of Interest

The authors declare no conflicts of interest.

## Peer Review

The peer review history for this article is available at https://www.webofscience.com/api/gateway/wos/peer‐review/10.1111/joor.14048.

## Data Availability

The data that support the findings of this study are available on request from the corresponding author.
